# Effects of dendritic core–shell glycoarchitectures on primary mesenchymal stem cells and osteoblasts obtained from different human donors

**DOI:** 10.1186/s12951-015-0128-y

**Published:** 2015-10-08

**Authors:** Stefan Lautenschläger, Christin Striegler, Olga Dakischew, Iris Schütz, Gabor Szalay, Reinhard Schnettler, Christian Heiß, Dietmar Appelhans, Katrin S. Lips

**Affiliations:** Laboratory for Experimental Trauma Surgery, Justus-Liebig-University Giessen, Schubertstr. 81, 35392 Giessen, Germany; Leibniz-Institut für Polymerforschung Dresden e.V., Hohe Str. 6, 01069 Dresden, Germany; Department of Trauma Surgery, University Hospital of Giessen-Marburg GmbH, Campus: Giessen, Rudolf-Buchheim-Str. 7, 35392 Giessen, Germany

## Abstract

The biological impact of novel nano-scaled drug delivery vehicles in highly topical therapies of bone diseases have to be investigated in vitro before starting in vivo trials. Highly desired features for these materials are a good cellular uptake, large transport capacity for drugs and a good bio-compatibility. Essentially the latter has to be addressed as first point on the agenda. We present a study on the biological interaction of maltose-modified poly(ethyleneimine) (PEI-Mal) on primary human mesenchymal stem cell, harvested from reaming debris (rdMSC) and osteoblasts obtained from four different male donors. PEI-Mal-nanoparticles with two different molecular weights of the PEI core (5000 g/mol for PEI-5k-Mal-B and 25,000 g/mol for PEI-25k-Mal-B) have been administered to both cell lines. As well dose as incubation-time dependent effects and interactions have been researched for concentrations between 1 μg/ml to 1 mg/ml and periods of 24 h up to 28 days. Studies conducted by different methods of microscopy as light microscopy, fluorescence microscopy, transmission-electron-microscopy and quantitative assays (LDH and DC-protein) indicate as well a good cellular uptake of the nanoparticles as a particle- and concentration-dependent impact on the cellular macro- and micro-structure of the rdMSC samples. In all experiments PEI-5k-Mal-B exhibits a superior biocompatibility compared to PEI-25k-Mal-B. At higher concentrations PEI-25k-Mal-B is toxic and induces a directly observable mitochondrial damage. The alkaline phosphatase assay (ALP), has been conducted to check on the possible influence of nanoparticles on the differentiation capabilities of rdMSC to osteoblasts. In addition the production of mineralized matrix has been shown by von-Kossa stained samples. No influence of the nanoparticles on the ALP per cell has been detected. Additionally, for all experiments, results are strongly influenced by a large donor-to-donor variability of the four different rdMSC samples. To summarize, while featuring a good cellular uptake, PEI-5k-Mal-B induces only minimal adverse effects and features clearly superior biocompatibility compared to the larger PEI-25k-Mal-B.

**Electronic supplementary material:**

The online version of this article (doi:10.1186/s12951-015-0128-y) contains supplementary material, which is available to authorized users.

## Background

Dendritic polymers feature versatile molecular architectures with multifunctional properties [1–6]. Tailored by the fabrication of the dendritic scaffold, the resulting dendritic polymers can provide multifunctional key features such as high water-solubility [7], complexing properties [8], conjugation properties [9, 10], self-assembling properties [11–13] and various biological properties [1–16]. In result complex dendritic structures have been impressively used in the field of biomedical applications over the last decade.

In line with this, first trials have been undertaken with dendritic polyamine scaffolds based on various (hyper-)branched poly(ethylene imines) (PEI) to use them as non-viral vectors for small-interfering-(si)-RNA [8, 17] and DNA [17–20]. PEI nanoparticles show on the one hand high cellular uptake rates and high transfection efficacies while on the other hand certain negative effects of viral-vectors like for example immune responses might be reduced or even not present. Initial enthusiasm cooled down a little bit because of problems that occur with the naked, unmodified PEI-nanoparticles. Negative effects are reported to be mainly high toxic effects due to positive charges and intercalation with DNA [18, 21], also the generation of reactive oxide species (ROS) is discussed [22–24]. Therefore approaches to enhance biocompatibility and cellular uptake have been undertaken. For example polyglutaminic acid-chains [25], oligosaccharide units [26, 27] or different other modifications [28, 29] have been introduced to better shield the PEI-core from the biological environment.

Especially, dendritic glycopolymers based on PEI or poly(propylene imine) cores exhibited their medical, pharmaceutical and biological potential, for example, as drug-delivery-system (DDS) [30–33], polymeric therapeutics and diagnostics [18, 21, 34–36], and artificial tubulating agents [22, 37]. The different interaction features of the dendritic glycopolymers against drug molecules, peptides, oligo-/polynucleic acids, proteins and various cells can be considered as highly adaptable. This key pivotal property is preferentially characterized by the multivalent interaction properties of the oligosaccharide architectures. This key property is further governed by the size and shape of the dendritic scaffold and (surface) charge of dendritic glycopolymers. Thus, it gives us the chance to capture new biomedical research topics with these highly adaptable dendritic glycopolymers.

However the careful evaluation of newly designed materials is crucial. In vitro trials are the first set of experimental methods available to investigate combinations of living cells and newly designed materials. Of course, as it is well known from biological systems, that a large donor-to-donor variety of cellular response on external influences exist. Commercial available cells are mostly available from one single donor only and cannot sufficiently account for that variety. Mesenchymal primary stem cells, harvested from the reaming debris (rdMSC) of different donors [38, 39], are suitable to overcome that limitation. In consequence the aim of this study was to carefully investigate the influence of the maltose-modified PEI nanoparticles against different rdMSC, harvested from multiple healthy donors. Furthermore to evaluate their value as potential drug delivery system the cellular uptake has been looked into. The experimental setup to investigate the given task consisted of various microscopy techniques and different quantitative assays.

## Methods

**Fig. 1:**
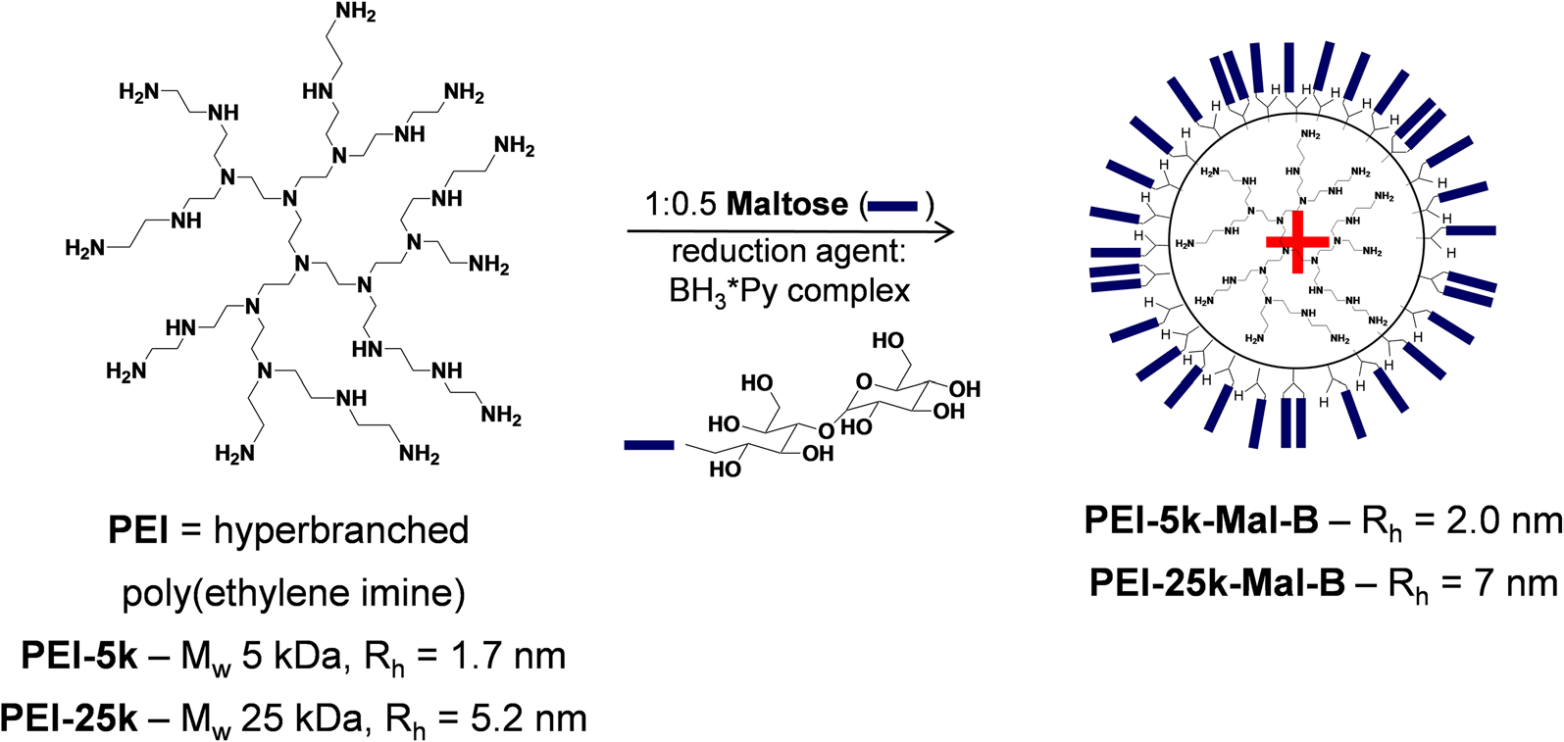
Synthetic approach of cationic dendritic core–shell glycoarchitectures PEI-5k-Mal-B and PEI-25k-Mal-B [26, 27]. Due to different sizes of hyperbranched poly(ethylene imine) cores final dendritic core–shell glycoarchitectures provide different hydrodynamic radii (R_h_) [26, 27]

Two dendritic core–shell glycoarchitectures (Fig. 1) with different molecular weights of the poly(ethylene imine) (PEI) core (25,000 g/mol, defined as PEI-25k-Mal-B and 5000 g/mol, defined as PEI-5k-Mal-B) have been investigated. The dendritic core–shell particles were synthesized by reductive amination of PEI in the presence of minor maltose. The molar ratio between amino groups of PEI and maltose is 1–0.5. Further details of the synthetic approach can be taken from previously described approach by Appelhans et al. [26]. Both PEI were obtained from commercially available source [Lupasol G100 for PEI-5k-Mal-B and Lupasol WF for PEI-25k-Mal-B by BASF SE (Ludwigshafen, Germany)]. The used dendritic core–shell glycoarchitectures (Fig. 1) are characterized by the molecular structure B where after the reductive amination of PEI the previous peripheral or primary amino groups are converted into preferential secondary amino groups possessing one chemically attached maltose unit besides minor tertiary amino groups bearing two chemically attached maltose units (Fig. 1) [26]. Both PEI-Mal-B macromolecules possess a lower cationic surface charge or charge density than their parental PEI [26, 27, 33, 40]. Therefore, dendritic core–shell glycoarchitectures [40] can be considered as weakly cationic and spherical polyelectrolyte [26] macromolecules with size dimensions of about 4 nm for the smaller nanoparticle PEI-5k-Mal-B (Fig. 1) and of about 14 nm for the larger nanoparticle PEI-25k-Mal-B (Fig. 1).

Further details of the charge density are presented in the Additional file 1. To emphasize that glycoarchitecture B based on PEI-5k and PEI-25k core molecules is a weakly cationic polyelectrolyte, a comparison of the established glycoarchitectures A–C [26] is presented as pH dependent measurements. One can recognize in both cases that the charge density, determined by polyelectrolyte titration experiments that glycoarchitecture B (Fig. 1) has lower cationic charge as found for glycoarchitecture C (possessing lower degree of attached maltose) [26] starting from isoelectrical point to lower pH values (Additional file 1: Figures S2, S3). The molecular weight of both glycoarchitectures were determined as described in a previous paper [26]: PEI-5k-Mal-B of about 18,000 g/mol and PEI-25k-Mal-B of about 38,100 g/mol.

Experimental description for carrying out DLS and polyelectrolyte titration experiments are presented in the Additional file 1.

### Mesenchymal stem cell cultures and osteogenic differentiation

Primary rdMSCs, labeled CC1, CC2, CC3 and CC4, have been harvested from reaming debris collected during trauma surgery at the university hospital Giessen. The exact procedure for obtaining vital rdMSC from reaming debris has been described by Wenisch et al. [39] and Trinkaus et al. [38]. In brief the harvested reaming debris has been incubated at 37 °C and under 5 % CO_2_ in Petri-dishes, with cell culture medium (Medium A) consisting of F12k (Gibco, Life Technologies, Carlsbad, USA) blended with 20 % fetal calf serum (PanSera ES, Pan Biotech, Aidenbach, Germany) and 1 % penicillin/streptomycin. After the rdMSCs grew out of the reaming debris they were harvested using 0.05 % Trypsin (Gibco, Life Technologies, Carlsbad, USA) and stored at liquid nitrogen temperature. Prior to the experiments the cells have been removed from their storage at liquid nitrogen temperature and were gently unfreezed. After thawing they were immediately transferred into cell culture bottles containing Medium A. Before seeded into 24 well plates for the experiments, the used rdMSC were passaged at least 2 times, not exceeding 3 times. The cell culture medium has been changed every fifth day. All experiments have been conducted in doubles and for all experiments 10,000 cells per cm^2^ have been seeded out either in 24-well plates (functional-assays) or in chamber slides (transmission electron microscopy and fluorescence microscopy). For our experiments four rdMSC lines have been used, all from healthy male donors, between 33 and 65 years.

To induce osteogenic differentiation in some of our experiments, Medium B, prepared as described by Trinkaus et al. [41], composed of Dulbeccos modified Eagles medium (Gibco, Life Technologies, Carlsbad, USA) low glucose, 0.05 mM ascorbic acid, 0.1 μM Dexamethasone, 10 % fetal calf serum, 10 mM β-glycerolphosphate and 1 % penicillin/streptomycin was added to the cells. The medium has been changed at least every fifth day.

### Determination of toxicity

To investigate dendritic core–shell glycoarchitectures´ toxic effects on rdMSC, 10,000 cells per cm^2^ have been seeded out in 24-well plates (Falcon Multiwell, Becton–Dickinson, Franklin Lakes, USA). For 1 day unmodified Medium A has been applied. On the second day the medium was replaced with Medium A containing each nanoparticle at a selected time using concentrations of 1 and 0.05 mg/ml. For each run the medium has been harvested after 24 and 72 h, respectively, and immediately used to conduct the Cytotox 96^®^ Non-Radioactive Cytotoxicity Assay (Promega GmbH, Mannheim, Germany) which measures the concentration of lactat dehydrogenase (LDH). The remaining cells, after washing 2 times in a 10 % phosphate buffered saline (PBS) solution, have been frozen at −80 °C. After 24 h the cells were unfrozen and 1 ml of 1 % Triton-X lysis buffer per well was added. After centrifuging of the lysates, 50 µl of each supernatant was treated according to the assay protocol of the Cytotox 96^®^ Assay. The total absorbance measured at 490 nm is proportional to the LDH concentration in the samples. To determine the cytotoxicity the measured LDH content in the medium was divided by total LDH content, determined by adding LDHmedium + LDHcell. All results were measured in triplicate. Quotients of >0.1 are considered to hint on toxic effects.

### Quantification of cell proliferation

The influence on cell proliferation after treatment with nanoparticles has been analyzed by again seeding out 10,000 cells/cm^2^ in 24 well plates. After 1 day the unmodified medium A was replaced with medium A containing nanoparticle concentrations of either 1 mg/ml or 0.05 mg/ml of PEI-25k-Mal-B or PEI-5k-Mal-B. Three different endpoints, 7, 21 and 28 days, were investigated. After washing the cells in a 10 % PBS solution, 250 μl of 1 % Triton X was added. The lysates were centrifuged and 3 × 10 μl of each supernatant transferred to three different wells of a 96-well plate. We used the DC-Protein-Assay (Bio-Rad Laboratories, Hercules, USA) to quantify cell numbers. The prepared 96-well plates were treated according to the assay protocol. 25 ml of reagent A and 200 ml of reagent B were added. After 15 min of incubation at room temperature the absorbance at 750 nm has been recorded. For each used rdMSC the assay has been calibrated by seeding out 10,000, 20,000, 40,000 and 80,000 cells per Well, performing the DC protein assay, subsequently followed by performing a linear regression.

### Alkaline phosphatase assay and von-Kossa-staining: influence of nanoparticles on osteogenic differentiation

The alkaline phosphatase is the leading enzyme of the bone metabolism. Its concentration in the cells hints on the differentiation behavior of osteogenic cell cultures. 10,000 cells per cm^2^ have been seeded out in 24 well plates. To induce osteogenic differentiation medium B (described above) has been used. Additionally to the unmodified Medium B, we assembled medium B containing nanoparticle concentrations of 1, 20 and 50 μg/ml of PEI-25k-Mal-B or PEI-5k-Mal-B. Every fifth day, the medium has been changed. Each modification has been tested in doubles for 7, 21 and 28 days. After each incubation time the medium has been collected and the cells have been washed in 10 % PBS solution. Afterwards, 250 ml 1 % Triton X lysis buffer has been added and the lysates have been centrifuged. According to the test protocol of the used SensoLyte pnPP ALP Assay (AnaSpec, Fremont, USA) 3 × 10 μl supernatant of each sample were added to separate wells of a 96-well plate. After addition of the assay buffer and incubation for 45 min at 37 °C the absorbance at 405 nm has been recorded. The absolute amount of ALP has been referenced on the cell number obtained by conducting the DC-Protein-Assay.

In addition, to prove synthesis of mineralized matrix, von-Kossa stained samples with osteogenic differentiated cells have been manufactured [42]. The von Kossa stained samples have been semi-automatically histomorphometrically evaluated using ImageJ software [43, 44]. For each sample 5 sections, each 1.1 mm × 1.5 mm, have been evaluated. The images have been converted to black and white, the threshold was set and the mineralized area has been measured. The mineralized area of each MSC sample has been normalized to the respective non nanoparticle treated reference sample.

### Light microscopy

In addition all samples have been checked multiple times, using a 090-135.002 light microscope (Leica GmbH, Wetzlar, Germany) equipped with a Nikon Ds-Fi1 camera (Nikon, Duesseldorf, Germany).

### Fluorescence microscopy (CLSM)

In addition to the unmarked nanoparticles, Rhodamine B labeled PEI-25k-Mal-B and PEI-5k-Mal-B were used to proof cellular uptake of the particles. Intracellular features like DNA or actin filaments have been stained by 4′,6-diamidino-2-phenylindol (DAPI, Carl Roth GmbH, Karlsruhe, Germany) for the DNA or phalloidin coupled TRITS dye (Sigma-Aldrich, St. Louis, USA) for the actin filaments. 10,000 Cells per cm^2^ were seeded into chamber slides. After 1 day treatment with Medium A + nanoparticles (1 mg/ml) the cells were washed and stained according to the protocols. For imagining a TCS SPS (Leica GmbH, Wetzlar, Germany) microscope has been used, excitation and detection wavelengths have been chosen as follows: DAPI: excitation 405 nm, detection 460 nm, phalloidin-TRITS: excitation 561 nm, detection 577 nm, Rh-PEI-Mal-B: excitation 405–498 nm, detection 520 nm.

### Transmission electron microscopy (TEM)

Cells have been seeded out in chamber slides and were incubated for 60 min and 24 h with a concentration of 1 mg/ml of each used nanoparticle. At the endpoints the cells have been washed with 0.1 M PBS buffer and fixed with yellow-fix mixture (2 % paraformaldehyde, 2 % glutaraldehyde, 0.02 % picric acid mixed with 0.1 M phosphate buffer). After this step fixation in 1 % osmium tetroxide in 0.1 M sodium cacodylate buffer took place. Dehydration steps, involving washing in rising concentrations of alcohol, followed consecutively. The final steps of sample preparation involved embedding in EPON resin, ultra-thin-cutting (80–100 nm), staining with uranyl acetate and lead citrate and investigation in a LEO912 (Carl Zeiss AG, Oberkochen, Germany) transmission electron microscope.

### Statistics

Statistical calculations were done using SPSS V21 (IBM, Armonk, USA). For each endpoint either Kruskal–Wallis calculations or, if Gaussian distribution was existent, ANOVA with Tukey Post-Hoc tests were calculated. The level of significance was set to p ≤ 0.05 and the confidence interval to 95 %. The boxplots show the standard error (SE) as the width of the box, the whiskers cover the whole range of measured values. The bar displays the median while the diamond marks the intersection.

## Results

### Toxicity of nanoparticles

**Fig. 2:**
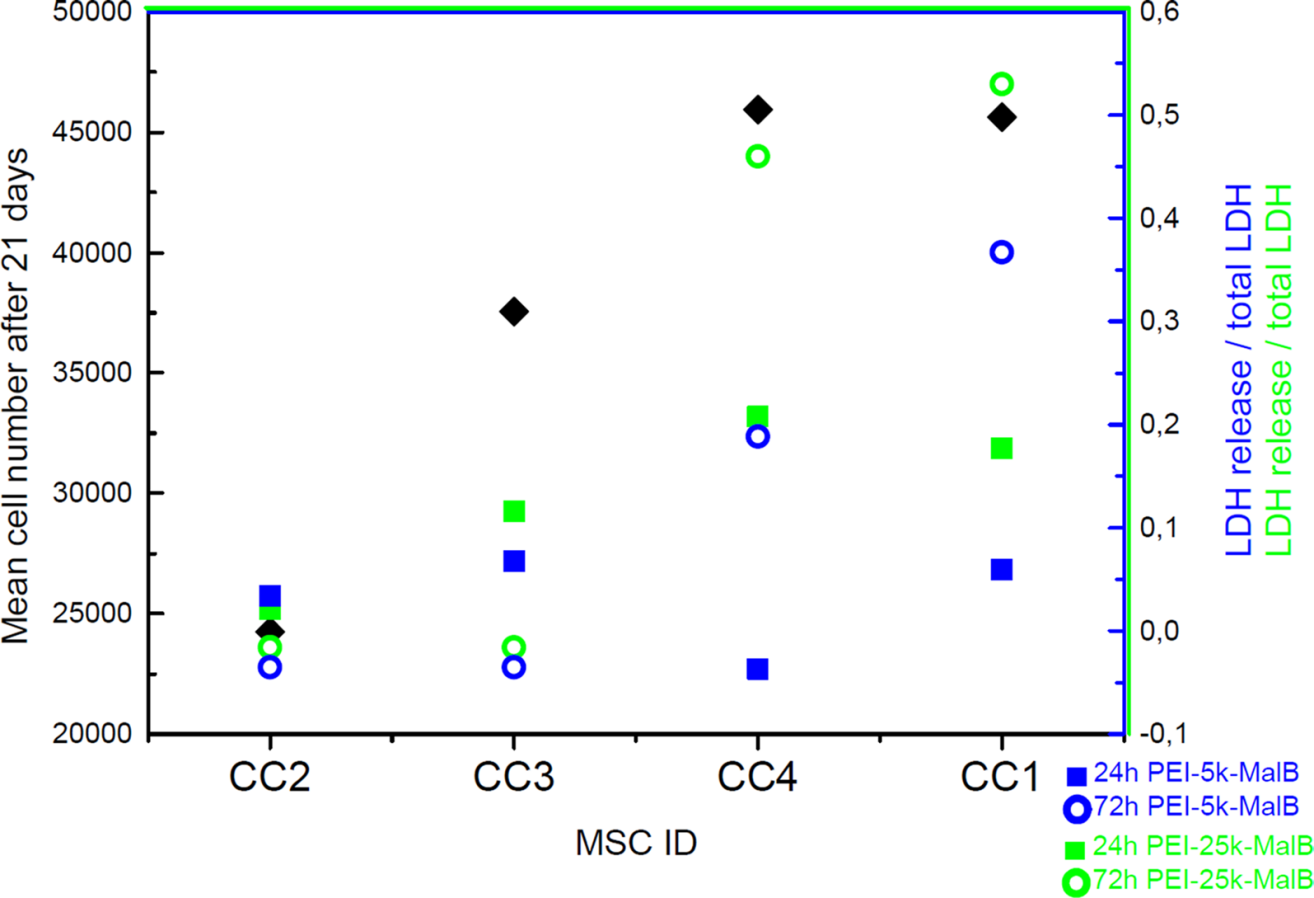
Mean cell number after 21 days (*black diamonds*) and cytotoxicity for 24 h (*squares*) and 72 h (*circles*) for each cell culture incubated with 1 mg/ml PEI-25k-Mal-B. Fast proliferating rdMSC exhibit a pronounced toxic response when treated with nanoparticles

**Fig. 3:**
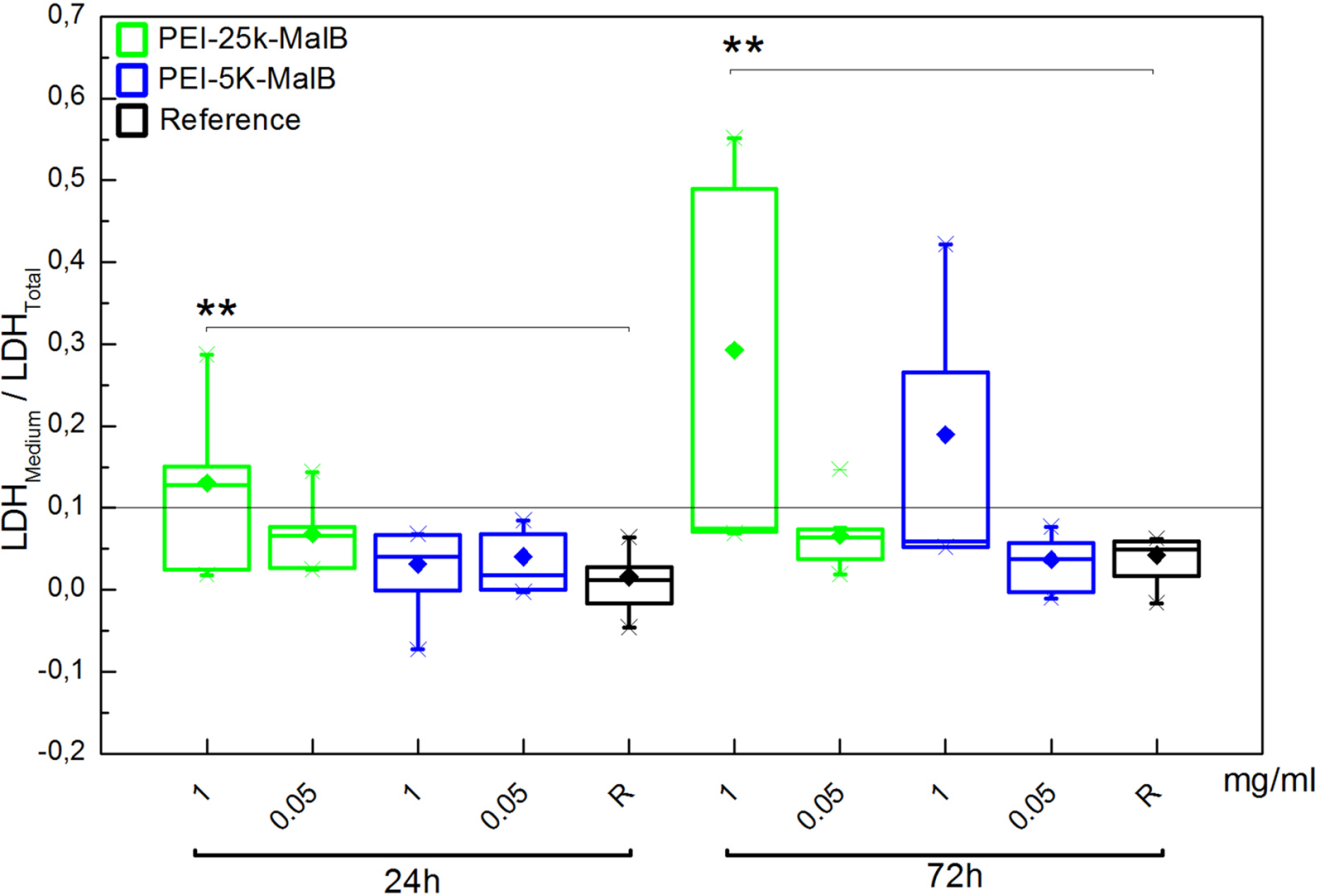
Cytotoxicity of plain nanoparticles for two different endpoints and with two concentrations of each nanoparticle, determined by LDH assay. For PEI-25k-Mal-B is toxic at its higher concentration, while PEI-5k-Mal-B shows no significant toxic response. The width of the *boxes* is the SEM while the *diamonds* represent the intersection (**p ≤ 0.01)

Possible toxic effects of the used materials were investigated for both nanoparticles (PEI-25k-Mal-B and PEI-5k-Mal-B) in different runs with two incubation times (24 and 72 h) and two nanoparticle-concentrations (1 and 0.05 mg/ml). For every separate condition cells have been seeded out in doubles. The observed toxic effects of the nanoparticles seem to be on the one hand dependent on the used concentrations and on the other hand on the molecular weight of the PEI core. In addition a strong donor-dependency for adverse effects of both nanoparticles from the used cell culture can be observed (Fig. 2). However generally the smaller PEI core for PEI-5k-Mal-B induces significant lower toxic effects for all incubation times, concentrations and rdMSC. While for both endpoints the toxicity of the higher concentration of PEI-25k-Mal-B compared to the untreated reference is significant (p = 0.006 for both) (Fig. 3).

### Influence on proliferation

**Fig. 4:**
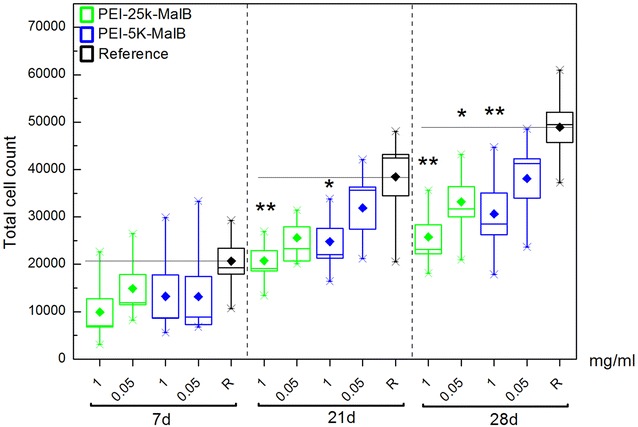
Total cell count after 7, 21 and 28 days, with different concentrations and types of nanoparticles. The *horizontal black line* for each endpoint marks the mean of the reference samples. Differences in cell count are significant after 21 days for 1 mg/ml of each nanoparticle, after 28 days for PEI-25k-Mal-B both tested concentrations result in reduced cell numbers (**p ≤ 0.01, *p ≤ 0.05)

Long term investigations with time scales of 7, 21 and 28 days were conducted. Concentrations of 1 and 0.05 mg/ml for both nanoparticles have been investigated. The DC-protein-assay was used to quantify total cell numbers. The total cell numbers for all four used rdMSC-lines combined have been evaluated and are displayed in Fig. 4. The long term experiments yield in reduced cell numbers when adding nanoparticles, statistically significant for high nanoparticle concentrations of both particles and incubation times of 21 and 28 days. In the case of PEI-25k-Mal-B a 28 days incubation period with the lower nanoparticle concentration also resulted in significantly reduced cell numbers. For 7 days the statistical calculations are not significant but exhibit a certain trend towards reduced cell numbers for experiments with nanoparticles as well.

### Osteogenic differentiation

**Fig. 5:**
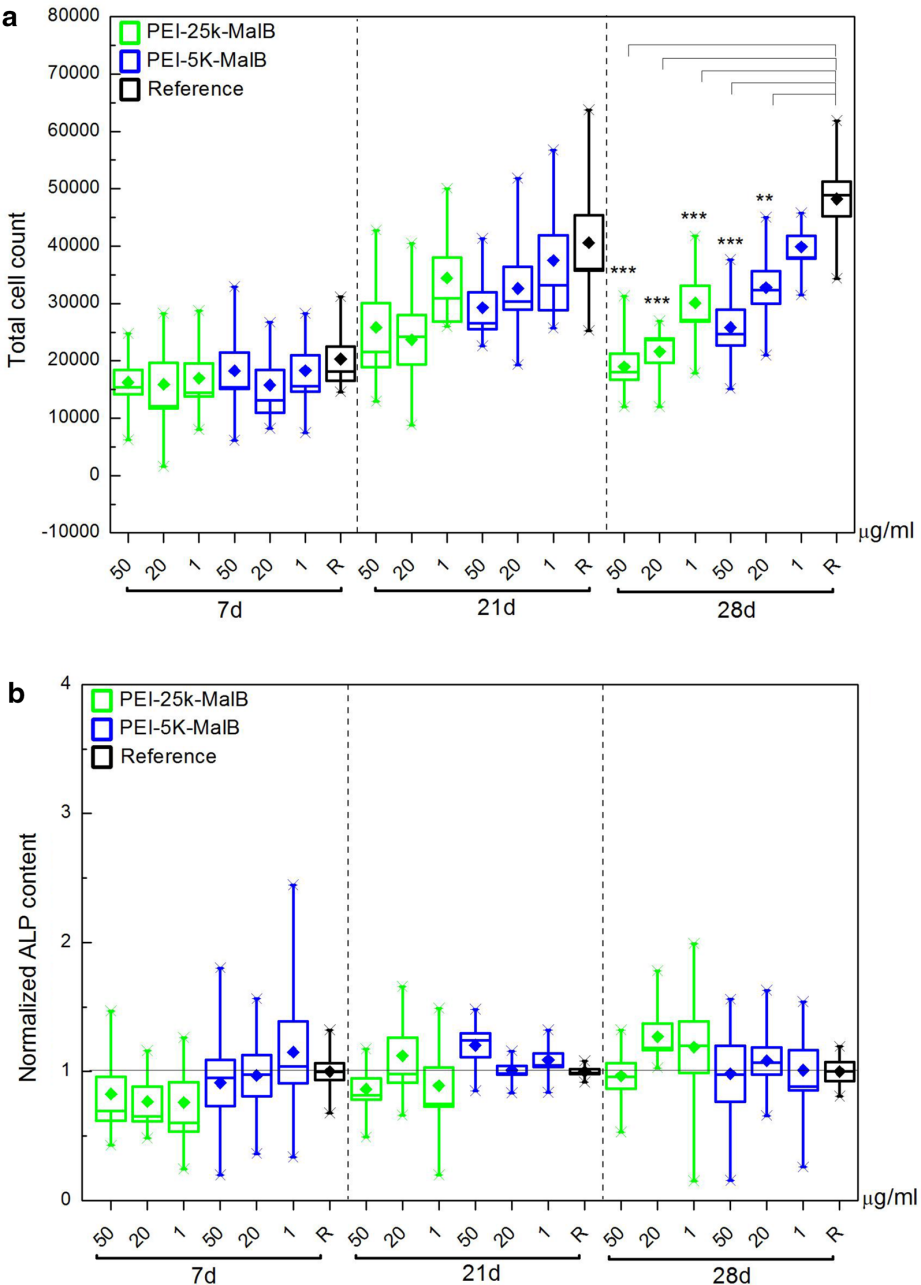
**a** Total cell count of osteogenic differentiated cells determined by DC-protein-measurements. After 28 days a significant reduction in cell count is observed for all concentrations of PEI-25k-Mal-B and for the higher and the intermediate concentration of PEI-5k-Mal-B (***p < 0.001, **p < 0.01). **b** Normalized ALP content per cell of rdMSC differentiated to osteoblasts and incubated with different concentrations of both nanoparticles. The measured total ALP concentration has been divided by the cell count. As reference for normalization the respective control samples for each endpoint have been used. No influence of nanoparticles on the ALP concentration per cell has been detected

**Fig. 6:**
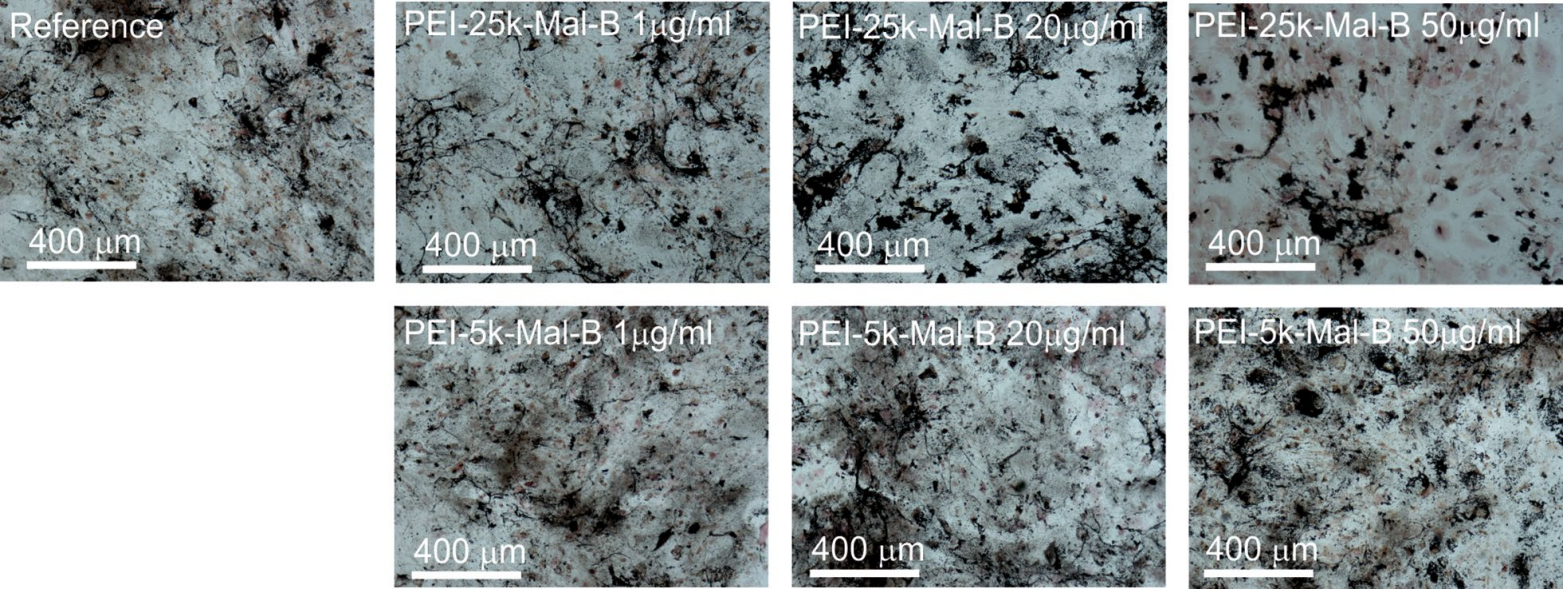
Light microscopy images of von-Kossa stained osteogenic samples, incubated with or without addition of nanoparticles. The deep *black* structures represent mineralized matrix

The influence of the nanoparticles on the osteogenic differentiation of rdMSC has been investigated by measuring the ALP content of the cells after 7, 21 and 28 days as endpoints (Fig. 5b). In consideration of the results of the proliferation tests (Fig. 4) decreasing concentrations of dendritic core–shell glycoarchitectures (0.05 mg/ml = 50, 20 and 1 μg/ml) have been selected. As demonstrated in Fig. 5b, the particles, no matter at what concentration, induce no change of the measured normalized ALP concentration while the proliferation of osteogenic differentiated cells is reduced (Fig. 5a). The latter one is comparable to the results obtained from the rdMSC-experiments (Fig. 4). In addition von-Kossa stained samples of osteogenic differentiated rdMSC, with and without nanoparticles, have been prepared. Figure 6 shows large structures of mineralized matrix, deep black in the image, which hint on excessive production of mineralized matrix and thus on a successful osteogenic differentiation for all investigated conditions, weather incubated with or without nanoparticles. The histomorphometric evaluation of the von-Kossa stained samples exhibited no statistically significant deviations from the reference samples for all investigated conditions. However, while not significant, for the larger particle PEI-25k-Mal-B possibly a trend towards higher mineralization can be found. For all conditions the addition of nanoparticles did not lead to reduced osteogenesis.

### Cellular uptake (fluorescence microscopy)

**Fig. 7:**
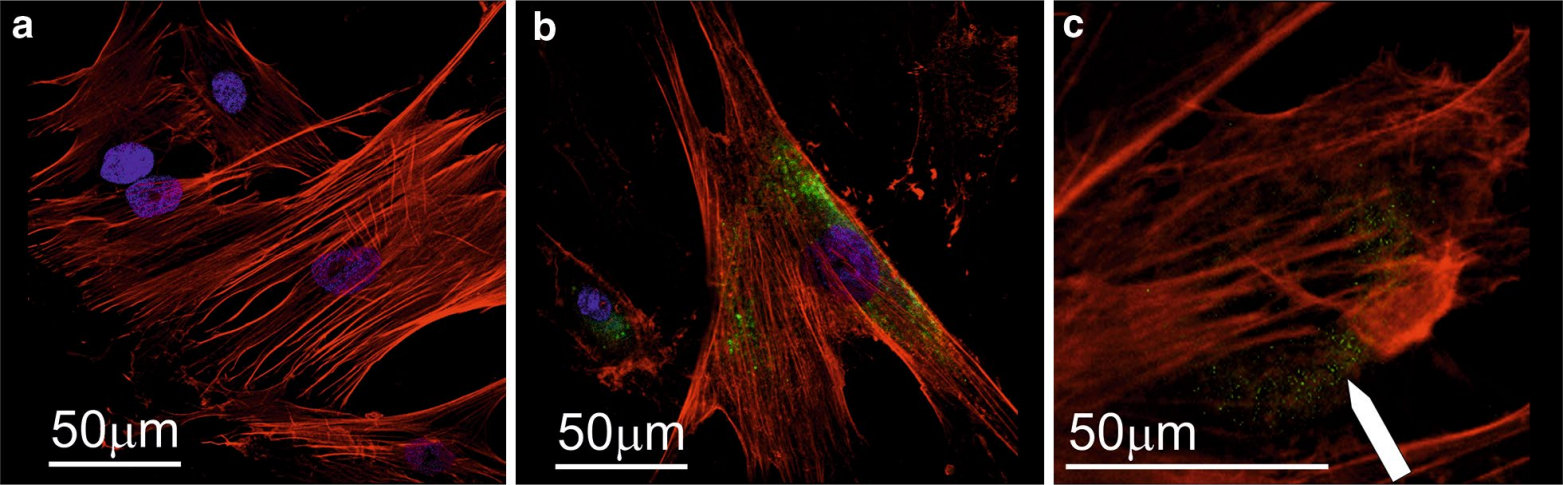
CLSM images of cell cultures treated with rhodamin B marked nanoparticles (*green*). The nucleus has been DAPI dyed (*blue*), the actin filaments were stained with phalloidin coupled TRITS (*red*). **a** Displays untreated reference, only the nucleus and the actin-filaments can be observed. **b** Rhodamin B labeled Rh-PEI-5k-Mal-B nanoparticles, surrounding the nucleus. **c** The rhodamin-marked nanoparticles Rh-PEI-25k-Mal-B feature a perinuclear distribution, in this sample the nucleus has not been stained

To proof the cellular uptake of PEI-5k-Mal-B and PEI-25k-Mal-B by CLSM, both were labeled by Rhodamine B (Rh-PEI-5k-Mal-B or Rh-PEI-25k-Mal-B, green) and administrated to rdMSC for 24 h with concentrations of 0.1 mg/ml. The nucleus and the actin filaments were stained with either DAPI (blue) or Phalloidin coupled TRITS (red). The reference (Fig. 7a) shows no signs of nanoparticles. As shown in Fig. 7b, sample has been treated with Rh-PEI-5k-Mal-B where the nanoparticles feature a peri-nuclear distribution. For samples incubated with Rh-PEI-25k-Mal-B (Fig. 7c), again a peri-nuclear particle distribution can be found, in this case the nucleus has not been stained (Fig. 7c).

### Cell morphology

**Fig. 8:**
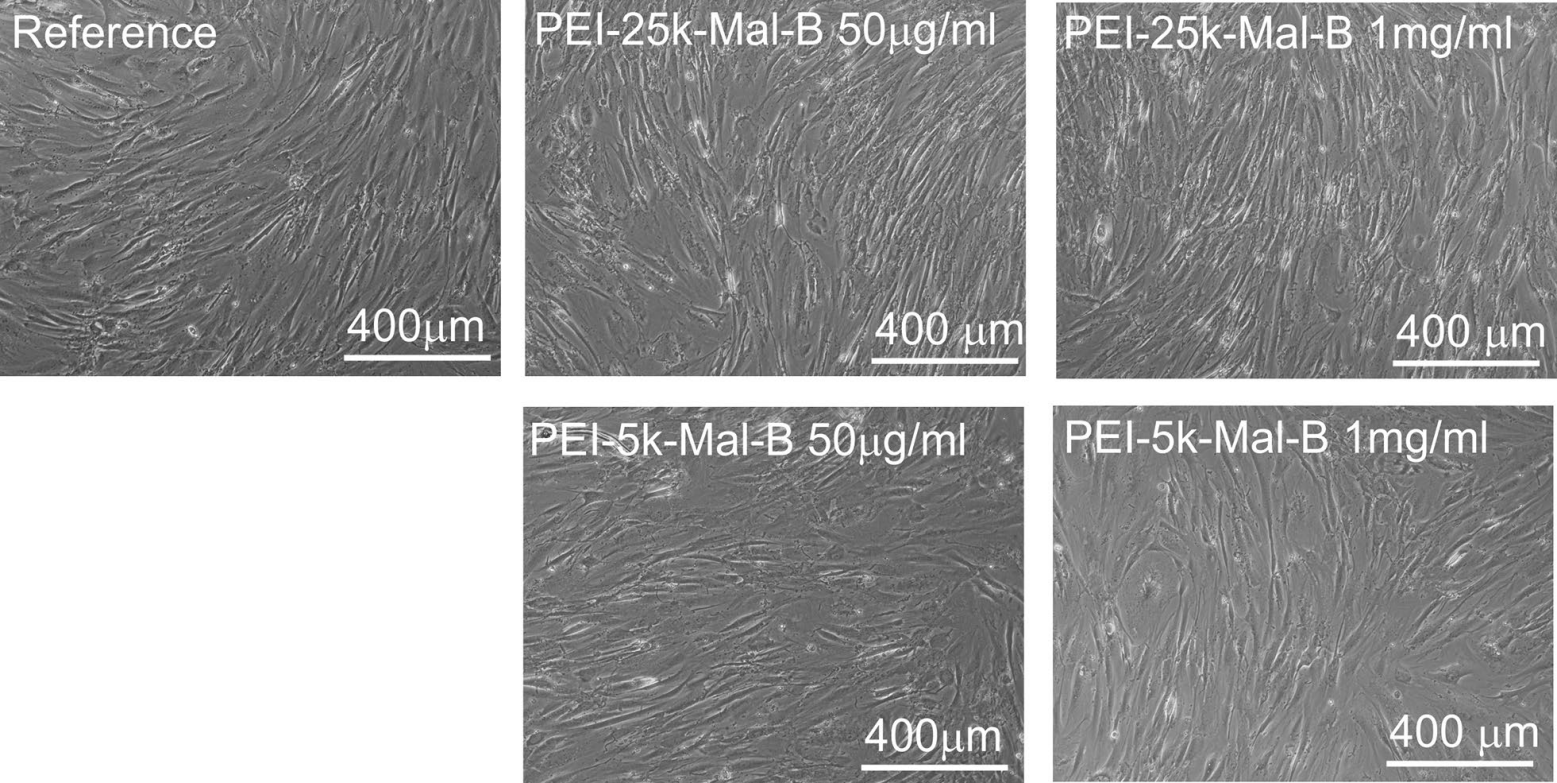
Light microscopy images, taken for endpoints of 72 h. rdMSCs treated with nanoparticles and the reference. For samples treated with nanoparticles cells going into apoptosis (for PEI-25k-Mal-B) or reduced cell numbers (both nanoparticles) can be found

The development of cell morphology of rdMSC (Fig. 8) was investigated by light microscopy when administrating nanoparticles PEI-25k-Mal-B and PEI-5k-Mal-B to cells for 72 h. In particular cell cultures treated with larger PEI-25k-Mal-B in both concentrations exhibit signs of cellular damage like for example cellular debris, floating free in the medium. Compared to the reference samples incubated in unmodified Medium A, even this short time exposure seems to reduce the cell count of nanoparticles-incubated rdMSC too.

**Fig. 9:**
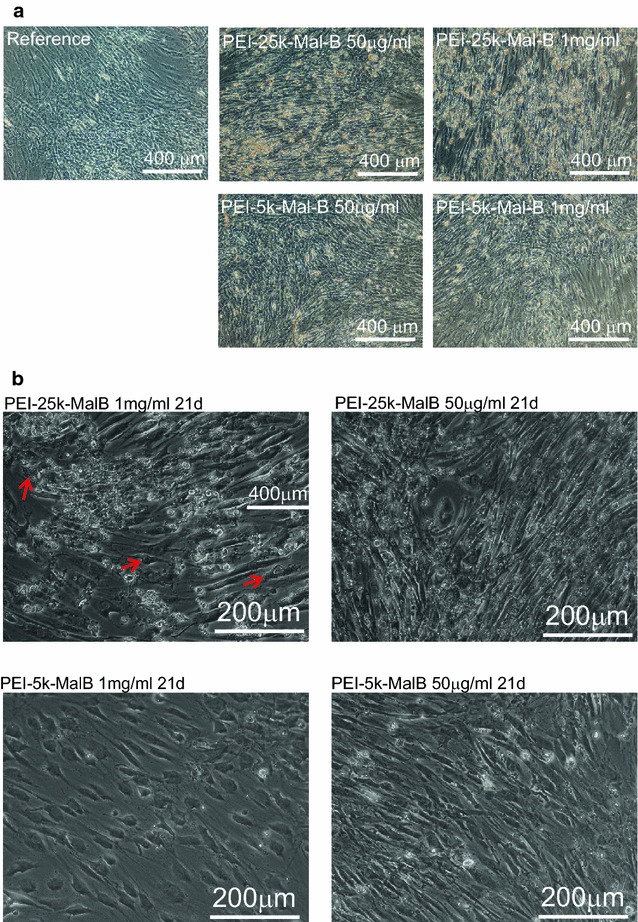
**a** Light microscopy images for long-term trials with endpoint 21 days. Compared to the reference especially the rdMSC treated with PEI-25k-MAL-B exhibit damaged cells and free floating cell debris, while rdMSC incubated with PEI-5k-Mal-B show less signs of damage. **b** Light microscopy images with higher magnification of cells treated with nanoparticles for 21 days. Especially PEI-25k-Mal-B results in large amounts of cellular debris (*white* structures) and damaged cells (*arrows*), while PEI-5k-Mal-B doesn’t harm cells in that way

Additionally, light microscopy images obtained from long-term trials after 21 days are shown in Fig. 9. The cells of the untreated reference rdMSC are vital and confluent cells. No signs of damage, reduced viability or distorted morphology can be found. In contrast to this reduced cell counts are observable for both nanoparticles, while for rdMSC treated with PEI-25k-Mal-B signs of cellular damage and death, as for example irregular nuclei, distorted cell shapes or free floating cell debris, can be detected. PEI-5k-Mal-B, in both concentrations, induces far less damage and cell debris.

**Fig. 10:**
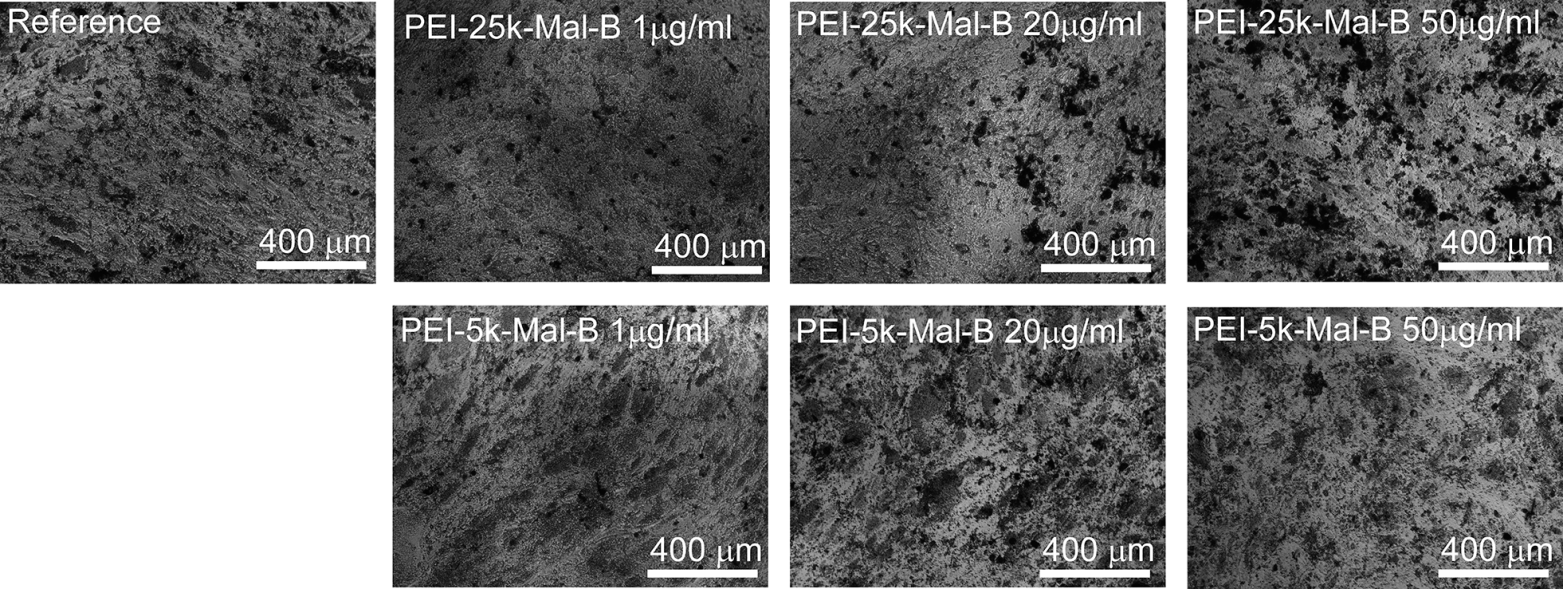
Light microscopy images of osteogenic differentiated cells for the endpoint 28 days. From the images no influence on differentiation of cells from nanoparticles can be distinguished, cell debris can be found for PEI-25k-MAL-B treated samples

Moreover, morphologic features of cells with osteogenic differentiation are also displayed as light microscopy images in Fig. 10. Blocks of mineralized matrix can be seen in all light microscopy images, especially in the von-Kossa stained ones (Fig. 6). Due to the high mineralization it is difficult to distinguish cells or parts of them. However again the rdMSC samples treated with the intermediate (20 µg/ml) and high (50 µg/ml) PEI-25k-Mal-B concentration exhibit free floating cell debris (black dots and circular structures) that hint on toxic effects induced by the nanoparticles.

On smaller scales, a deeper insight into the cell morphology in the presence of nanoparticles can be gained by transmission electron microscopy (TEM). As incubation times 60 min and 24 h have been selected. The shorter one to research the uptake and the longer one to investigate possible early cytotoxic effects. All experiments have been carried out using nanoparticle concentrations of 1 mg/ml.

**Fig. 11:**
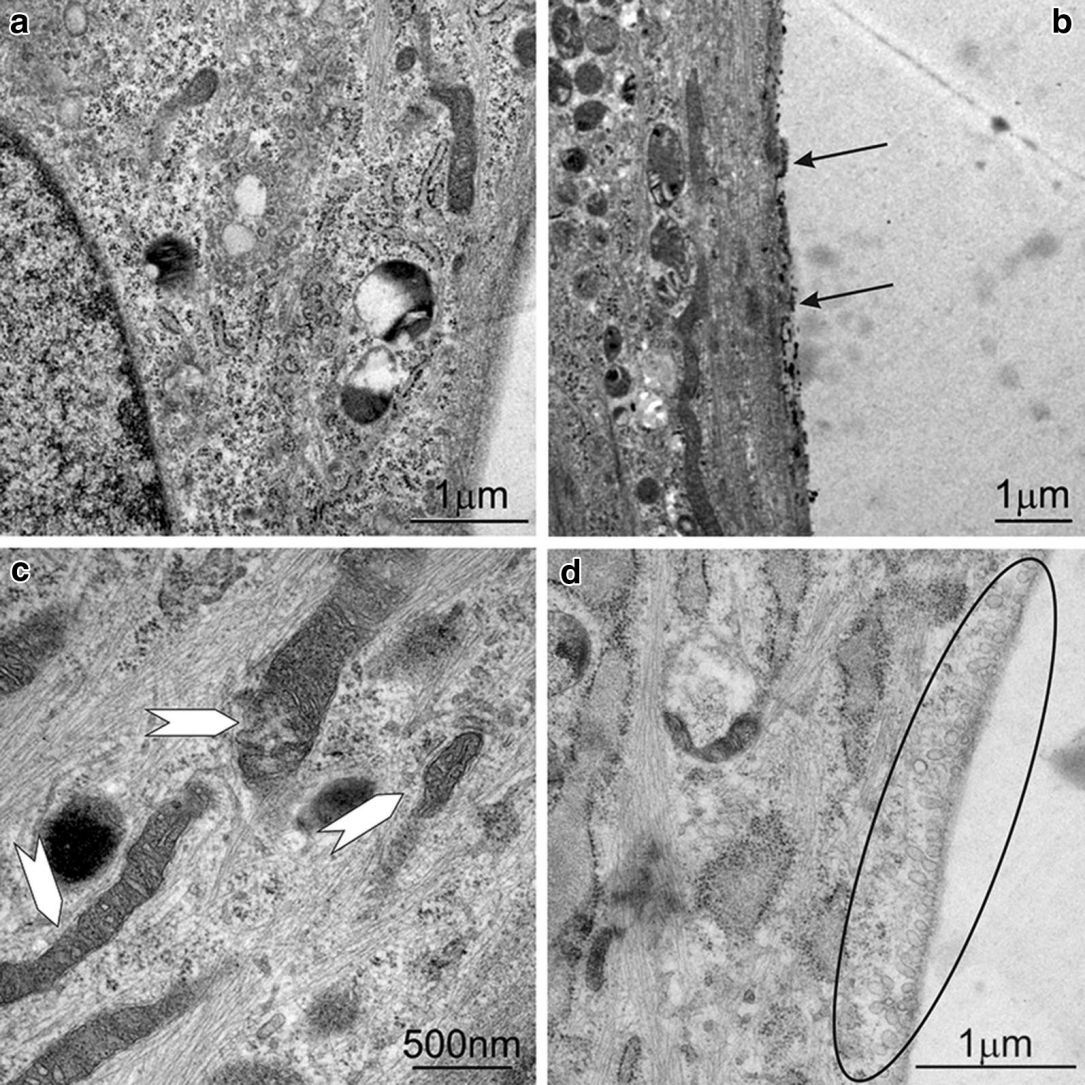
TEM images of **a** reference without nanoparticles, **b** PEI-25k-MAL-B treated cells, small structures (marked with *arrows*) sticking on the cell membrane can be seen **c** PEI-25k-MAL-B treated cells, endpoint 24 h, the *arrows* mark signs of mitochondrial destruction **d** PEI-5k-MAL-B treated samples, vesicular uptake of nanoparticles can be seen

An untreated reference cell can be seen in Fig. 11a. No extracellular particles are present, their intracellular structures are intact. No sign of cellular damage can be observed. In contrast, PEI-25k-Mal-B treated cells, incubated for 60 min, feature small nanometer-sized particles, attached to the cell membrane surface (hints by arrows in Fig. 11b). Additionally to this, one can impressively recognize the toxic effects of PEI-25k-Mal-B on rdMSC viability after 24 h. Mitochondrial damage (see Fig. 11c) is exclusively available for PEI-25k-Mal-B, the inner structure of the mitochondria is distorted and the mitochondrial membrane is beginning to disintegrate. Moreover, vesicular structures in the area of rdMSC membrane can be also visualized when applying smaller nanoparticles PEI-5k-Mal-B. This structural detail during the cellular uptake process of nanoparticles is presented in Fig. 11d.

## Discussion

Dendritic glycopolymers, and in particular oligosaccharide-modified PEI nanoparticles, provide a large variety of desired features for applications in biomedicine [45]. However one has to weigh the pros and cons of their usage and carefully investigate and analyze their positive and negative influences on cells and organism. Prior to in vivo applications of these nanoparticles an extensive and diligent in vitro testing is necessary for acquiring their biological properties against rdMSC and in differentiation process of rdMSC to osteoblasts.

To gain maximal results from the experimental setups it is crucial to conduct on the one hand experiments with a variety of cells from different donors and on the other hand to expand the experimental time-scales up to 28 days. To the best of our knowledge, such long-term in vitro experiments have not been undertaken so far, neither with plain rdMSC nor with osteoblasts. In detail, one of our most important tasks was to determine any donor dependent variability in the impact of the used nanoparticles on the different rdMSC lines. For primary rdMSC it has been reported that there is a large donor-to-donor variance for example for the proliferation of cells [46] or the influence of growth inhibitors on cells [47]. Our experiments yielded that for as well untreated cells as ones incubated with nanoparticles the speed of proliferation shows a large donor-dependent variability. A very interesting finding here is that there is a connection between the speed of proliferation and the observed toxicity. The fast proliferating cell cultures (CC1 and CC4) reveal a considerably higher toxic response compared to the low-proliferating CC2 and the intermediate one CC3 (Fig. 2).

While cells incubated with PEI-5k-Mal-B showed no microscopic signs of toxicity, in most the cases the mitochondrial system of cells treated with PEI-25k-Mal-B was negatively affected by these larger glycoarchitectures (Fig. 11c). One possible origin of toxicity from core–shell glycoarchitecture nanoparticles is the generation of reactive oxygen species (ROS) [48] that interfere with the mitochondrial system [49] and drive the cell into apoptosis. Comparable results from literature up to now are mainly available for different dendritic structures. First Kuo et al. [23] found out that cationic parental poly(propylene imine) (PPI) dendrimers influence the generation of ROS in human macrophages. Additionally they proposed that the equilibrium between ROS generation and detoxification, which is essentially for macrophages, is disturbed and the generation of ROS is increased in such cells. Janaszewska et al. [24] investigated the influence of unmodified PPI, maltotriose modified PPI and poly(amidoamine) (PAMAM) dendrimers on human and hamster ovarian carcinoma cells. Again the generation of ROS has been identified as one main source of toxicity. In this context the cationic open shell PPI glycodendrimers also provides toxicity behavior at higher concentration. Calarco et al. [22] reported about reactive oxygen species (ROS) that play a role in PEI induced cell damage while the surface-modified PEI nanoparticles also outlines reduced genotoxicity. Together with the reports from literature, our TEM images (Fig. 11c) suggest that for administrating PEI-25k-Mal-B mitochondrial damage, caused by the generation of ROS, drives the cell into apoptosis. Additional to the inter-species dependent variability in the toxic response of the cells [24], we found a significant donor-to-donor variable toxicity for our rdMSC. This implies a new key aspect which has to be considered in future in vitro studies with oligosaccharide-modified PEI nanoparticles, but may be also for other types of nanosized carrier systems.

The second important task to investigate was the long term influence of the used nanoparticles on a time scale up to 28 days. Most experiments preferentially investigated incubation times of ≤72 h [22, 25, 27, 50, 51] to describe undesired effects such as toxic effects and reduced proliferation already to be detectable after 24 h, but also at longer incubation times of 48 and 72 h. However none of the experiments extended their incubation times up to a point when a steady state of negative effects has been reached. In consequence we extended the endpoints of our proliferation and differentiation experiments up to 28 days. Early toxic effects are preferentially observable on a time scale between 24 and 72 h and can be quantified by the LDH release and optical microscopy. In particular for the larger particle PEI-25k-Mal-B, we found that undesired toxic effects began to occur after 24–72 h incubation time. This is in good agreement with other authors [22, 25, 27, 50, 51]. However, the long-term experiments after 21 and 28 days visualized by light microscopy gave a deeper insight on the biological action of both core–shell glycoarchitectures on rdMSC (Fig. 9). In line with this countable (toxic) effects of both core–shell glycoarchitectures can be quantified by the determination of reduced cell numbers after 21 and 28 days by using DC protein assay (Fig. 4). For both nanoparticles it gives a marginal or slight difference in the cell numbers after 21 and 28 days. The light microscopy images show lots of cellular debris floating free around the cells. Therefore, we assume that the reduced cell number in our long term experiments is also caused by the generation of ROS followed up by apoptosis. Of course the presence of other negative influences like for example direct interference with nucleic acids, denaturation of proteins or inflammatoric response cannot be completely excluded at the moment. Thus, the observed toxic effects of core–shell glycoarchitectures were mainly dependent on the molecular weight of the particle and the concentrations used. Both nanoparticles have the same degree on maltose shell decoration. It mainly implies that this parameter has a lower influence on the biological action of both nanoparticles. In principle both core–shell glycoarchitectures are not completely comparable with other PEI structures, but it gives a good agreement with the results reported by Fischer et al. [18]. They also describe a molecular-weight-dependent behavior for their naked, unmodified PEI molecules. In our case negative influences on cell count and proliferation of the smaller particle PEI-5k-Mal-B are minimal, if not completely absent, while the negative influences are even more pronounced for the larger particle PEI-25k-Mal-B. Similar results obtained from in vivo experiments in mice conducted by Gutsch et al. [33] who described that PEI-5k-Mal-B features a superior biocompatibility due to their reduced surface charge, while unmodified PEI nanoparticle with molecular weight of 5000 g/mol (PEI-5k) outlines enhanced toxic effects. In line with this denser oligosaccharide shell on PEI-5k also provides enhanced biocompatibility [33]. In addition to this Höbel et al. [27] used SKOV3 cells for testing the biocompatibility of oligosaccharide-modified PEI-5k and only found toxic effects almost exclusively for naked PEI-5k molecules. Overall the core–shell glycoarchitecture PEI-5k-Mal-B5k was successfully tested in various in vitro and in vivo experiments showing desired biocompatibility and carrier properties for various nucleic acid derivates [26, 27, 33]. Finally, one can conclude from these long-term results (21 and 28 days) that PEI-5k-Mal-B shows more promising biological effects/properties in the presence of rdMSC than the larger PEI-25k-Mal-B in our study. Moreover, in the course of short-term experiments (≤3 or 7 days), again PEI-5k-Mal-B is the most favorite of tested core–shell glycoarchitectures where the molecular weight of PEI core plays the most important key rule in the biological action of nanoparticles used here in our study.

**Fig. 12:**
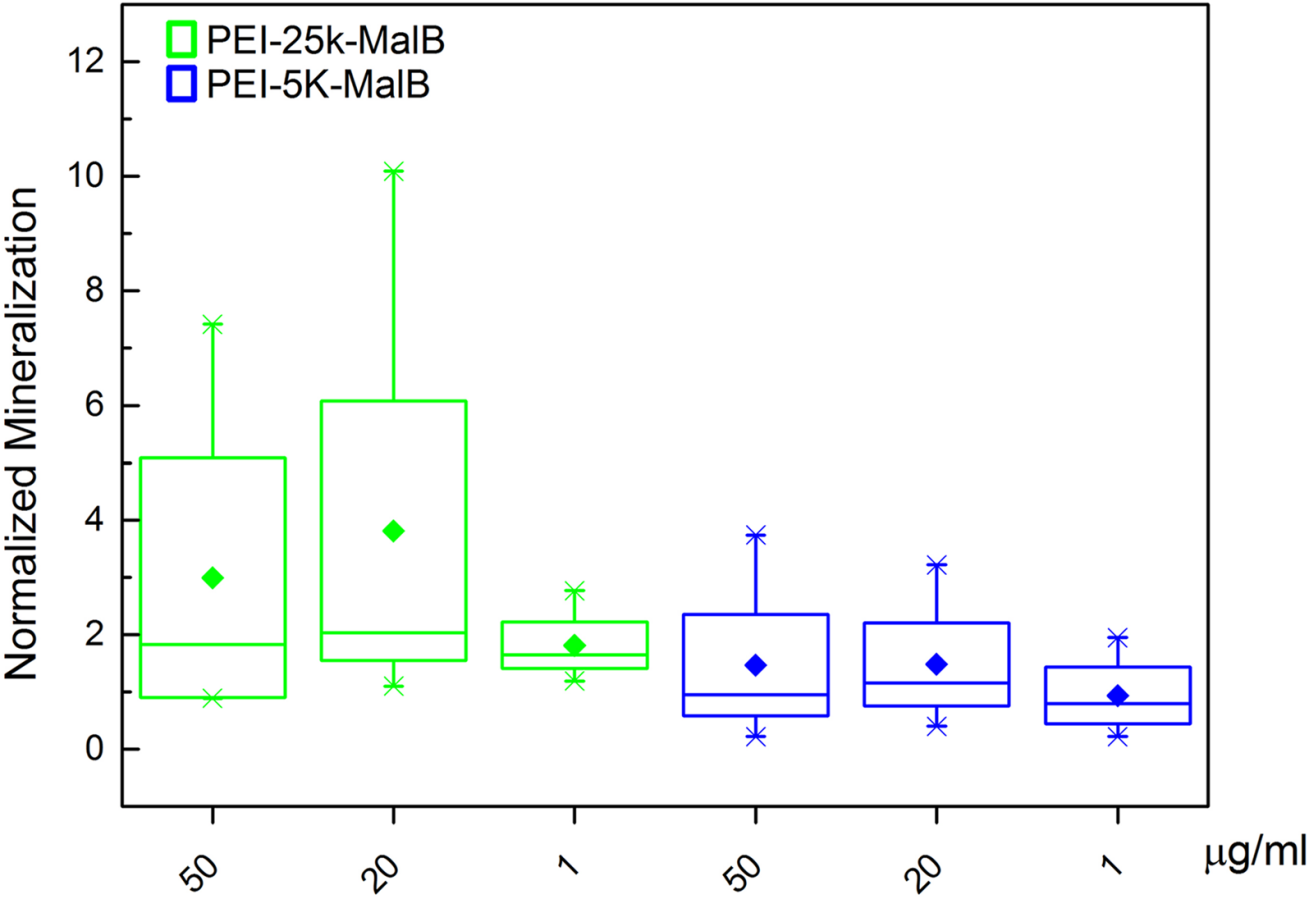
Statistical, histomorphometric evaluation of the von-Kossa stained samples. The area of mineralized matrix is referenced to the respective reference sample for each condition. (For PEI-25k-Mal-B p = 0.163, for PEI-5k-Mal-B p = 0.511)

Despite the long-term effects of cationic core–shell glycoarchitectures on cell viability and proliferation, the differentiation of rdMSC to osteoblasts is not influenced by the nanoparticles (Fig. 5). In opposite to (slightly) lower cell number in the presence of nanoparticles (Fig. 5a), the ALP content of the cells (Fig. 5b) and the production of mineralized matrix (Figs. 6, 12) in the absence and presence of nanoparticles do not differ statistically significant. Further studies if (oligo-)maltose-modified-PEI nanoparticles with higher molecular weights influence osteogenesis in a positive way, as it might be suggested by the histomorphometric evaluation of the von-Kossa stained samples, might have a chance of success if we first succeed in further improvement of biocompatibility. Certainly none of the used (oligo-)maltose-modified-PEI nanoparticles in our case influences the capability of osteogenic differentiation in a negative way. This behavior has been reported for other nanoparticle-systems, for example, using low concentrations of Ag nanoparticles [52]. Again, only the further study by light microscopy will give a clear key feature of both core–shell glycoarchitectures on differentiated rdMSC (Fig. 10). Only in the case of PEI-25k-Mal-B cell morphology is clearly influenced showing the presence of cell debris. This enables us to conclude that the cell function after the differentiation of rdMSC to osteoblasts, checked by the ALP content, is not the deciding experiment (Fig. 5b) to do final conclusion on the long-term biological action of both core–shell glycoarchitectectures. Only the combination of light microscopic study (Fig. 10) and determination of DC-protein measurement (Fig. 5a) and ALP determination (Fig. 5b) gives us a deeper view on the biological action of both nanoparticles where the smaller PEI-5k-Mal-B is the most promising core–shell glycoarchitecture in these experiment series. With this in mind, one may postulate that the core–shell glycoarchitecture PEI-5k-Mal-B with the smaller PEI-5k core can be used as drug delivery system for treating bone disease in locally applied therapy.

## Conclusion

In summary we have demonstrated that maltose-modified poly(ethylene imine) (PEI)nanoparticles with PEI-5k and PEI-25k core feature a good uptake in primary mesenchymal stem cells. They do not negatively interfere with the differentiation capabilities of those, but induce different toxic and proliferation-inhibiting effects. The later ones are mainly tailored by the molecular weight of PEI core and the maltose shell modification of each PEI core. For the larger core–shell glycoarchitectures PEI-25k-Mal-B effects on the mitochondrial system is clearly visible by TEM images. Due to large differences in the interaction between cells and nanoparticles donor-to-donor variability of the used rdMSC has to be taken into account.

The most important breakthrough of this study was to evaluate the influence of incubation time (3, 21 and 28 days) of nanoparticles on the proliferation of rdMSC. To detect toxic effects of administered nanoparticles it seems crucial to use at least endpoints of 3 days considered as short-term experiments. Here, only first side effects on cell proliferation can be determined. Negative effects on the cell proliferation can be reliably detected after 21 days, considered as long-term experiments. Finally the particle with the lower molecular weight of the PEI-5k core, PEI-5k-Mal-B, features superior biocompatibility compared to those of PEI-25k-Mal-B. For PEI-5k-Mal-B lower early toxic effects and only slight effects on cellular proliferation are detectable in short- and long-term experiments. The cellular integrity stayed intact in rdMSC treated with this particle. This makes PEI-5k-Mal-B a suitable candidate as drug delivery vehicle for future in vitro and in vivo studies.

## Additional file

10.1186/s12951-015-0128-y Additonal information on the investigated (oligo-)maltose-modified PEI nanoparticles.

## Abbreviations

DAPI: 4′,6-diamidino-2-phenylindol
ALP: alkaline phosphatase
DC: detergent compatible
DFG: Deutsche Forschungsgemeinschaft (German research foundation)
LDH: lactate dehydrogenase
PEI-Mal: maltose-modified poly(ethyleneimine)
rdMSC: reaming-debris-mesenchymal-stem-cells
PAMAM: poly(amidoamine)
PPI: poly(propylene imine)
ROS: reactive oxygen species
Rh: rhodamine
Ag: silver
SFB: Sonderforschungsbereich (collaborative research center)
TRITS: tetramethylrhodamine B isothiocyanate
TEM: transmission electron microscopy
TrkB: tryosine receptorkinase B
