# Supplementary material for: Effects of dendritic core–shell glycoarchitectures on primary mesenchymal stem cells and osteoblasts obtained from different human donors
Source: J Nanobiotechnology. 2015 Oct 8;13:65. doi: 10.1186/s12951-015-0128-y (PMC4597403; doi:10.1186/s12951-015-0128-y)
Supplement: Supplementary file 1 — 10.1186/s12951-015-0128-y Additonal information on the investigated (oligo-)maltose-modified PEI nanoparticles. [file 12951_2015_128_MOESM1_ESM.docx]

**Supporting Information**

***Polyelectrolyte titration experiments***

Charge densities (q) of maltose-modified PEI were determined by polyelectrolyte titration in a particle charge detector combined with 702 SM Titrino. Solutions of low molecular weight poly(ethylene sodium sulfonate) (PES-Na) or poly(diallyldimethylammonium chloride) (PDADMAC) were used as titrants for cationic and anionic systems, respectively. q (meq/g) was calculated according to the following formula: q=C_titrant_V_titrant_/Vm, where C_titrant_ is the concentration of titrant (meq/L), V is the volume of titrated solution (mL), V_titrant_ is the equivalent titrant volume (mL), and *m* is the content of polyelectrolyte in titrated solution (g/L).

***Dynamic light scattering (DLS)***

To characterize the particle size and size distribution, dynamic light scattering (DLS) measurements were carried out at 25 °C at a fixed angle of 173° using the Nano Zetasizer (Malvern), equipped with a He–Ne laser (4 mW) and a digital autocorrelator. The observed data was analyzed very carefully. Thus only measurements with a good fit and an exponential graphic representation were considered here. The particle size distribution was determined using a multimodal peak analysis by intensity, volume and number after one. The zetasizer was also used for electrophoretic experiments to determine the electrophoretic velocity of dendritic glycopolymers. The velocity of macromolecules in an electric field, using 120 V/cm, was measured by laser Doppler anemometry employing the He/Ne laser. The apparent electrokinetic potential (zeta-potential, ζ) values were calculated from the complex velocity according to the Smoluchowski equation. Concentration of glycoarchitecture solution was 5µM/L.

**Size dimension of dendritic glycopolymer determined by DLS**

**Fig. SI-1.** Volume plot of dendritic glycopolymers PEI-5k-Mal-B and PEI-25k-Mal-B obtained by DLS measurements using concentration of 5µM/L of each dendritic glycopolymer.

**pH-dependent charge density and zeta potential of PEI-5k-Mal-B and PEI-25k-Mal-B**

**Fig. SI-2**. pH-dependency of charge density for PEI-5k, PEI-5k-Mal-A, PEI-5k-Mal-B and PEI-5k-Mal-C (concentration of polymers 0.5mg/ml) at a rate of Coulomb per gram determined by polyelectrolyte titration experiments.

**Fig. SI-3**. pH-dependency of charge density for PEI-25k-Mal-A, PEI-25k-Mal-B and PEI-25k-Mal-C (concentration of polymers 0.5mg/ml) at a rate of Coulomb per gram determined by polyelectrolyte titration experiments.

**Fig. 4-SI.** pH-dependent zeta potential of dendritic glycopolymers PEI-5k-Mal-B and PEI-25k-Mal-B 5µM/L of each dendritic glycopolymer.

From the pH-dependent polyelectrolyte titration experiments and pH-dependent zeta potential measurements following points can be extracted: Firstly, results from polyelectrolyte titration experiments allow the possibility to determine the complete charge density of dendritic glycopolymers´ architecture used in this study. It is recognizable that the larger PEI-25k-Mal-B outlines a larger charge density (**Fig. SI-3**) than for PEI-5k-Mal-B (**Fig. SI-2**) along decreasing pH values. This implies that the high number of N atoms in PEI-25k is responsible for the high charge density over the complete molecular architecture in PEI-25k-Mal-B. On the other hand pH-dependent zeta potential measurements imply the determination of the surface charge of dendritic glycopolymers at which the presence of additional ions partially influence the zeta potential of both dendritic glycopolymers PEI-5k-Mal-B and PEI-25k-Mal-B in an applied electric field when considering zeta potential at pH <4. Thus, it is reasonable that no large difference is available between the cationic surface charges of both materials below pH 4, while smaller differences for the surface charge can be seen for pH values ≥ 4.

**Stability of PEI-25k-Mal-B over time**

**Fig. SI-5**. pH-stability of PEI-25k-Mal-B after different time periods. Various borate and phosphate buffers have been used for this study. Experiment series was repeated 3 times (concentration of PEI-25k-Mal-B: 0.5 mg/mL).
